# 

**Published:** 1916-07

**Authors:** 


### Contents

Event and Comment .......... 301
A New Method of Brushing Teeth .......... 304
The Dentist and the Cancer Problem .......... 308
The Evidence Speaks for Itself .......... 316
A Dental Efficiency Test .......... 320
A Few Remarks on Riggs Disease—Being an Excerpt from Some Correspondence .......... 323
The Relation of Amebiosis to Pyorrhea Alveolaris .......... 324
Musings of a Simpleton .......... 328
Liquid Petrolatum and Its Trademark Names .......... 330
Dental Laws and the State Board .......... 332
Indents .......... 335
Announcements .......... 343

